# Decoding the exosomal nucleic acid delivery system axis of macrophage autophagy and immune reprogramming via multi-omics analysis

**DOI:** 10.3389/fmolb.2025.1711082

**Published:** 2025-12-18

**Authors:** Zhoujun Zhu, Wei Xiang, Pengchao Zhang, Parhat Yasin, Xinghua Song

**Affiliations:** 1 Department of Joint Surgery, The Sixth Affiliated Hospital of Xinjiang Medical University, Urumqi, Xinjiang, China; 2 Department of Spine Surgery, The Sixth Affiliated Hospital of Xinjiang Medical University, Urumqi, Xinjiang, China

**Keywords:** autophagy, macrophage, miRNA-155, multi-omics, tuberculosis

## Abstract

**Background:**

MicroRNA-155 (miR-155) is a key regulator of macrophage function, and its abnormal expression is closely associated with the pathogenesis of tuberculosis (TB)—a disease where impaired macrophage autophagy weakens anti-mycobacterial immunity. Exosomes are promising nucleic acid carriers due to their biocompatibility and cell-targeting ability. Here, we constructed exosome-based miR-155 delivery systems (Exo-miR155-ago/Exo-miR155-antago; “ago” = agomir, a miR-155 agonist that enhances its expression; “antago” = antagomir, a miR-155 antagonist that inhibits its expression) to modulate macrophage autophagy and remold anti-TB immune responses.

**Methods:**

Exosomes were isolated from the supernatant of bone marrow mesenchymal stem cells using differential centrifugation. The miR155-5p agomir and antagomir were transfected into exosomes via the Exosome Transfection Kit, followed by co-incubation with macrophages. Transcriptomics and proteomics were employed to screen for differentially expressed genes and proteins. Western blot was employed to detect autophagy-related proteins and phosphorylated proteins in signaling pathways (p- denotes phosphorylation, a key post-translational modification regulating protein activity). Techniques including transmission electron microscopy (TEM), Monodansylcadaverine (MDC) staining, and reverse transcription-quantitative polymerase chain reaction (RT-qPCR) were applied to detect the autophagic level of macrophages.

**Results:**

Transcriptome sequencing identified 704 differentially expressed genes, with significant enrichment in TNF and NF-κB pathways, differential expression of NF-κB target genes (e.g., autophagy core gene Beclin1), and expression changes in key genes of the energy metabolism-related AMPK/mTOR pathway; proteomic analysis found 164 differentially expressed proteins, including key molecules of the “Pathogen Recognition-TLR4-NF-κB-Autophagy-Related Gene Transcription” pathway (TLR4, p-p65) and core proteins of the AMPK/mTOR pathway (p-AMPK, p-mTOR); functional verification showed the Exo-miR155-ago group had more autophagosomes (TEM), higher autophagic vacuole accumulation (MDC staining), upregulated mRNA/protein of autophagy-related molecules (LC3B, Beclin1), downregulated mRNA/protein of p62 (RT-qPCR/Western blot), activated p-p65 (NF-κB pathway), and increased p-AMPK with decreased p-mTOR (AMPK/mTOR pathway), and all results confirmed Exo-miR155-ago promotes macrophage autophagy via the synergistic effect of the two pathways.

**Conclusion:**

This study provides multi-omics evidence for autophagy modulation mediated by the exosomal nucleic acid delivery system, verifies that this system regulates macrophage autophagy by controlling the TLR4-NF-κB pathway and AMPK/mTOR pathway, and clarifies the application potential of this system in tuberculosis (TB) and other macrophage-associated.

## 1 Introduction

Tuberculosis (TB) is a global public health issue caused by infection with *Mycobacterium tuberculosis* (Mtb), posing a severe threat to human health (Wallis et al., 2023). According to the Global Tuberculosis Report 2023 released by the World Health Organization (WHO), there are over 10 million new TB cases worldwide each year, with mortality reaching the million level (Farhat et al., 2024). Among these, the incidence of drug-resistant tuberculosis (DR-TB) continues to rise, having become a key bottleneck restricting TB prevention and control (Saukkonen et al., 2025). Although anti-TB drugs and vaccines are currently available, the emergence of drug-resistant strains and the limitations of existing treatment methods have made the development of new therapeutic strategies with high efficacy, low toxicity, and anti-drug resistance a core topic in the global TB research field (Stoltz et al., 2025). As the “first line of defense” against intracellular pathogens in the body’s innate immune system, macrophages play a dual regulatory role throughout the entire process of Mtb infection (Bo et al., 2023). Mtb has evolved an extremely sophisticated intracellular immune escape mechanism: by releasing virulence factors, it interferes with the fusion of phagosomes and lysosomes, prevents phagosome acidification and the release of hydrolytic enzymes, while inhibiting the production of reactive oxygen species (ROS) in macrophages and inducing the secretion of anti-inflammatory cytokines (Kundu et al., 2021; Shariq et al., 2025). This creates an intracellular microenvironment conducive to its own survival, ultimately leading to the continuous colonization and proliferation of Mtb within macrophages, and the transformation of infection from the acute inflammatory phase to the chronic latent infection phase. This is also one of the core pathological mechanisms underlying the prolonged course and high recurrence rate of TB (Nasiri and Venketaraman, 2025). Based on this, it is crucial to explore methods for immune remodeling of macrophages colonized by Mtb.

Autophagy, a highly conserved “self-cleansing” mechanism in eukaryotic cells, involves the formation of autophagosomes with a double-membrane structure that enclose intracellular damaged organelles, misfolded proteins, and invading pathogens (Yamamoto and Matsui, 2024). These autophagosomes then fuse with lysosomes to form autolysosomes, and the enclosed substances are degraded by hydrolytic enzymes in lysosomes, thereby maintaining intracellular homeostasis (Liu et al., 2023). In macrophages infected with Mtb, the activation of autophagy has been confirmed as a key defense mechanism for the host against Mtb. After autophagy activation, autophagosomes in macrophages can specifically recognize macrophages occupied by Mtb and directly degrade intracellular Mtb through the autophagy-lysosome pathway (Mishra et al., 2025).

Exosomes are lipid bilayer vesicles with a diameter of approximately 30–150 nm, secreted by almost all cell types via the endosomal pathway (Zeng et al., 2023). In recent years, they have been widely regarded as next-generation drug/nucleic acid delivery carriers due to their unique biological properties (Sen et al., 2023). Compared with traditional delivery systems (such as liposomes and viral vectors), exosomes offer advantages including high biocompatibility, favorable targeting ability, and diverse loading capacities (Dilsiz, 2024). Previous studies have shown that loading anti-TB drugs (e.g., rifapentine) into macrophage-derived exosomes can significantly improve the enrichment efficiency of drugs at infection foci (Xiang et al., 2025). Moreover, the “exosome-miRNA delivery system” can carry specific miRNAs into infected macrophages to precisely regulate key intracellular signaling pathways, providing a novel technical carrier for regulating autophagy and enhancing anti-Mtb immunity in this study.

MicroRNAs (miRNAs) are a class of non-coding RNA molecules with a length of approximately 18–24 nucleotides. They regulate the stability or translation efficiency of target genes by binding to their mRNAs, thereby playing important roles in various physiological and pathological processes of cells (Winter et al., 2009). Among them, microRNA-155 (miR-155), a highly conserved pleiotropic miRNA, is widely involved in biological processes such as immune regulation, inflammatory response, cell proliferation, and apoptosis (Hu et al., 2022). Studies have shown that miR-155 plays a key role in macrophage polarization, cytokine secretion, and anti-pathogen immunity (Pasca et al., 2020). Particularly in Mtb infection, the expression level of miR-155 changes significantly, suggesting that it may play an important role in the host’s anti-TB immunity (Wang et al., 2013; Rothchild et al., 2016). In this study, miR-155 was used as the “cargo” for the construction of the exosome nucleic acid delivery system.

This delivery system aims to utilize the excellent biological properties of exosomes to accurately deliver target genes to macrophages, activate related metabolic and inflammatory pathways, and ultimately induce macrophage autophagy. In this way, it can remodel the immune escape effect caused by Mtb within macrophages, opening up a new avenue for the development of novel and efficient anti-TB therapeutic strategies.

## 2 Materials and methods

### 2.1 Isolation of exosomes, construction and characterization of the exosome nucleic acid delivery system

#### 2.1.1 Isolation of exosomes

Numerous studies have shown that rat bone marrow mesenchymal stem cell (BMSC)-derived exosomes possess significant advantages as miRNA delivery vectors, including high biocompatibility, low immunogenicity, and stable ability to cross biological barriers. Preliminary experiments comparing the exosome yields of rat, mouse, and human BMSCs demonstrated that rat-derived BMSCs exhibited the highest yield, thus we selected their exosomes as the delivery vector. Primary bone marrow mesenchymal stem cells (BMSCs) were isolated from the bone marrow of young Sprague-Dawley (SD) rats. The cells were cultured in an incubator at 37 °C with 5% CO_2_ and saturated humidity, using a medium containing 10% fetal bovine serum (FBS, Gibco, Thermo Fisher Scientific, Waltham, MA, USA, catalog #10099141C) and 1% penicillin-streptomycin. Before collecting the cell supernatant, the cells were transferred to exosome-free medium and cultured for 12 h to eliminate interference from serum-derived exosomes. The collected supernatant was subjected to differential centrifugation for exosome isolation, following these steps: Centrifugation at 300×g for 10 min to remove cells and large debris, with the supernatant retained; Subsequent centrifugation of the retained supernatant at 2000×g for 30 min to remove small debris and large vesicles, with the supernatant retained again; Further centrifugation of the supernatant at 10,000×g for 30 min to remove medium-sized membrane vesicles and protein aggregates, with the supernatant retained; Final ultracentrifugation of the supernatant at 100,000×g and for 70 min (All centrifugation steps were performed at 4 °C). The supernatant was discarded, and the exosome pellet was resuspended in an appropriate volume of phosphate-buffered saline (PBS). To purify the exosomes, ultracentrifugation was repeated under the same speed and temperature conditions. The final exosome pellet was resuspended in a small volume of PBS, which could be used for subsequent experiments or stored at −80 °C for later use.

#### 2.1.2 Construction of the exosome nucleic acid delivery system

**FIGURE 1:**
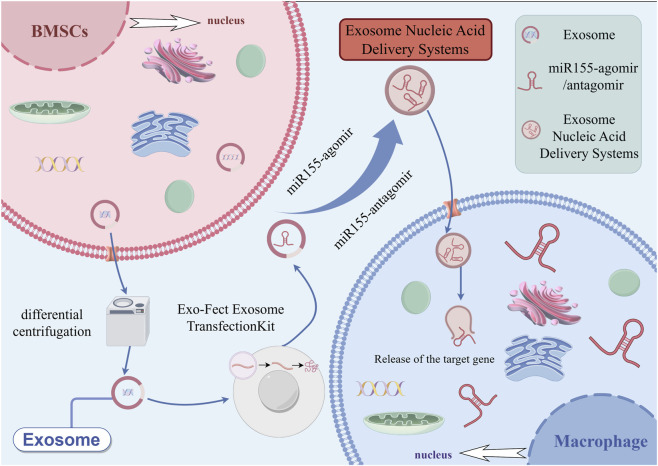
Schematic diagram of the preparation steps of exosome nucleic acid delivery system (Exo-miR155-ago/Exo-miR155-antago) and macrophage phagocytosis (By Figdraw, ID:UWSYle1686).

Literature has reported that mature miR155 is processed to generate two strands (miR155-5p and miR155-3p). Among them, the 5p strand is usually the “guide strand” that enters the RNA-induced silencing complex (RISC) to exert regulatory functions, while the 3p strand is mostly degraded as the “passenger strand” (Bartel, 2009). Furthermore, miR155-5p sequences from mice and rats are identical. Given that RAW264.7 cells are of mouse origin, we synthesized mouse-derived miR155-5p for transfection to ensure species consistency. Thus, mouse-derived miR155-5p was selected for subsequent experiments in this study. Experiments were performed using RNase-free 1.5 mL EP tubes (Axygen, Union City, CA, USA; Cat. No. MCT-150-C), and the following components were added to prepare a total transfection volume of 150 μL:

10 μL of Exo-Fect solution (Exo-Fect Exosome Transfection Kit, System Bioscience, Palo Alto, CA, USA), 20 μL of microRNA-155-5p-agomir (sense: UUAAUGCUAAUUGUGAUAGGGGU. Antisense: CCCUAUCACAAUUAGCAUUAAuu)/microRNA-155-5p-antagomir (Antisense: ACCCCUAUCACAAUUAGCAUUAA) (It was co-modified with 2′-O-methyl, phosphorothioate linkages, and cholesterol. 20 pmol, Tsingke Biotechnology Co., Ltd., Beijing, China), 70 μL of sterile 1×PBS, 50 μL of purified exosomes. The mixture was gently flicked or inverted three times for thorough mixing (vortexing was avoided). The transfection solution was incubated in a shaker at 37 °C for 10 min and then immediately placed on ice. Next, 30 μL of ExoQuick-TC reagent (provided in the kit) was added, and the tube was inverted six times for mixing (vortexing was avoided). After incubating the sample on ice for 30 min, it was centrifuged at 13,000–14,000 rpm at 4 °C for 3 min. The supernatant was discarded, and the exosome pellet was resuspended in 100 μL of 1×PBS. Finally, Exo-microRNA-155-5p-agomir (Exo-miR155-ago) and Exo-microRNA-155-5p-antagomir (Exo-miR155-antago) were obtained and stored at −80 °C for later use (The preparation procedure is shown in Figure 1).

#### 2.1.3 Characterization of the exosome nucleic acid delivery system

Particle Size Analysis: Nanoparticle Tracking Analysis (NTA) (Particle Metrix, ZetaView, Halbermoos, Germany) was used to determine the particle size of each sample group. Morphological Observation: Transmission Electron Microscopy (TEM) (HITACHI, HT 7800/HT 7700, Tokyo, Japan) was employed to observe the morphology of exosomes in each sample group. Typical exosomes exhibit a double-membrane structure and appear as cup-shaped or spherical vesicles. Western blot Analysis for Exosome Marker Proteins (GenScript Biotech Corporation, Nanjing, Jiangsu, China, catalog #M00521): The protein concentration of each sample was determined using the Bicinchoninic Acid (BCA) assay. The extracted exosomes were mixed with 5× protein loading buffer at a volume ratio of 4:1, heated in a boiling water bath for 10 min, cooled to room temperature, and then stored at −20 °C. The precast gel was fixed onto the electrophoresis tank (Beijing Liuyi Instrument Factory, Beijing, China, catalog #DYCZ-24DN), and electrophoresis buffer was poured into the reservoir. A micropipette was used to load the prepared protein samples and protein marker into the loading wells, with the total protein amount of each sample being 40 μg. After loading, electrophoresis was performed at a constant voltage of 180 V until bromophenol blue reached the bottom of the gel. After 40 min, the gel was removed, and the entire gel was directly used for membrane transfer. A PVDF membrane of the same size as the entire gel was cut; before transfer, the PVDF membrane was soaked in methanol for 30 s and then placed in equilibration buffer. The equilibrated PVDF membrane was placed, and the gel was lifted with a red plastic spatula and attached to the membrane in the marked order, followed by program setting. Membrane transfer conditions: for proteins below 20 kDa, set one cycle of 5 min each; for proteins between 20 kDa and 250 kDa, set two cycles of 5 min each; for proteins above 250 kDa, set three cycles of 5 min each. ECL development system (Affinity Biosciences, Cincinnati, OH, USA, catalog #KF8003): The PVDF membrane was soaked in TBST containing 5% non-fat milk (blocking buffer) and blocked on a shaker at room temperature for 2 h. The corresponding primary antibody was diluted with blocking buffer, and the PVDF membrane was immersed in the primary antibody incubation solution and incubated overnight at 4 °C. The PVDF membrane was thoroughly washed with TBST 5 times, 5 min each time. The corresponding HRP-conjugated secondary antibody was diluted 1:10000 with TBST, and the PVDF membrane was immersed in the secondary antibody incubation solution and incubated on a shaker at room temperature for 2 h. The PVDF membrane was thoroughly washed with TBST 5 times, 5 min each time. The enhancer solution and stable peroxidase solution in the ECL reagent were mixed at a ratio of 1:1, and the working solution was dropped onto the PVDF membrane. The PVDF membrane was exposed to X-ray film for signal detection and the gray value of the film was analyzed using ImageJ software. Details of the antibodies used are as follows: GAPDH (Sanying Biotechnology Co., Ltd., Wuhan, China; Cat. No.: #60004-1-Ig; Dilution ratio: 1:10000), TSG101 (Sanying Biotechnology Co., Ltd., Wuhan, China; Cat. No.: #28283-1-AP; Dilution ratio: 1:8000), CD9 (Abclonal, Woburn, MA, USA; Cat. No.: #A1703; Dilution ratio: 1:4000), CD63 (Sanying Biotechnology Co., Ltd., Wuhan, China; Cat. No.: #25682-1-AP; Dilution ratio: 1:1000).

### 2.2 Detection of transfection efficiency of exosome nucleic acid delivery system

#### 2.2.1 Analysis of transfection efficiency by flow cytometer

The exosome nucleic acid delivery system obtained through the above steps was used as the experimental group, and normal exosomes served as the blank control group. A FACScalibur flow cytometer (BD Biosciences, San Jose, CA, USA) was employed to analyze the transfection efficiency of Cy5-fluorescently labeled miR155-5p agonists and inhibitors in the exosome nucleic acid delivery system, so as to verify and confirm the successful construction of the exosome nucleic acid delivery system.

#### 2.2.2 Detection of miR155-5p expression by RT-qPCR

RNA in the exosome nucleic acid delivery system was extracted using the Trizol method. According to the operating procedures of the TaqMan MicroRNA Reverse Transcription Kit (Thermo Fisher Scientific, Waltham, MA, USA, Cat. No.: 4366596), This kit is mainly designed for the reverse transcription of specific miRNAs (e.g., miR-155-5p) and small nuclear RNAs (e.g., U6), this kit employs U6 as the linear gene-specific reverse transcription primer. RNA was reverse-transcribed into cDNA. With U6 as the internal reference (The stability of the reference gene was validated under our experimental conditions), the primers were designed and synthesized by Beijing Qingke Biotechnology Co., Ltd (primer sequences are shown in Table 1). Real-time fluorescent quantitative detection was performed in accordance with the operating procedures of TaqMan Universal Master Mix II (Thermo Fisher Scientific, Waltham, MA, USA). In addition, we detected the expression levels of miR155-5p in bone marrow mesenchymal stem cells (BMSCs) and their exosomes using the same method as described previously. The purpose is to clarify the expression level of endogenous miR155-5p in BMSC-derived exosomes, so as to exclude the impact of their endogenous miR155-5p on this study.

**TABLE 1:** Relevant primer sequences, including miR155-5P-RT, miR155-5P, U6, LC3B, p62, Beclin1, and GAPDH.

Gene name	Forward	Reverse
miR155-5P-RT	GTCGTATCGACTGCAGGGTCCGAGGTATTCGCAGTCGATACGACACCCCT	
miR155-5P	GCGGCTTAATGCTAATTGTGAT	ACTGCAGGGTCCGAGGT
U6-RT	AACGCTTCACGAATTTGCGT	
U6	CGCTTCGGCAGCACATATAC	TCACGAATTTGCGTGTCATC
LC3B	GCGGGTGATTATAGAGCGATACA	AAGCGCCGTCTGATTATCTTGAT
p62	CACAGGCACAGAAGACAAGAGTA	CCTGTAGATGGGTCCACTTCTTT
Beclin1	AAACTGGACACGAGCTTCAAGAT	CCATCCTGGCGAGTTTCAATAAA
GAPDH	ACTCTTCCACCTTCGATGCC	TGGGATAGGGCCTCTCTTGC

### 2.3 Phagocytosis detection of exosome nucleic acid delivery system

RAW264.7 is a widely used mouse macrophage cell line in *Mycobacterium tuberculosis* infection research, which exhibits clear responses to *Mycobacterium tuberculosis* infection (e.g., autophagy regulation) and has mature detection methods. The use of this cell line ensures the reliability and comparability of the results of this study with previous research. The macrophage cell used in this study was RAW264.7 line (Procell Life Science & Technology Co., Ltd., Wuhan, China; Cat. No.: CL-0190). To verify the phagocytosis of the exosomal nucleic acid delivery system by RAW264.7 cells, the exosomal nucleic acid delivery system labeled with PKH 67 (MCE, Cat. No.: 257277-27-3) was co-incubated with RAW264.7 cells in a cell incubator for 12 h, with a control group set up for comparison. After fixation with 4% paraformaldehyde, nuclear staining was performed (Servicebio, DAPI, Cat. No. G1012-100ML, Wuhan, China). The distribution of labeled exosomes in RAW264.7 cells among different groups (Blank group, Exo-miR155-ago group, Exo-miR155-antago group) was observed under a confocal microscope (Zeiss LSM 880, Oberkochen, Germany), and fluorescent quantitative analysis was conducted using ImageJ software.

### 2.4 *In vitro* and *in vivo* toxicity tests

The CCK-8 method was used for quantitative detection of the cytotoxicity of exosome nucleic acid delivery systems at different concentrations (Beyotime Biotechnology, Shanghai, China). Based on the CCK-8 assay results, an appropriate concentration of the exosome nucleic acid delivery system was selected for cell live/dead staining. Furthermore, we detected the proliferation (Ki67) and apoptosis (BCL-2/BAX) of RAW264.7 cells and RAW264.7 cells loaded with BMSC-derived exosomes using qRT-PCR, so as to clarify the effect of endogenous miR155-5p in BMSC-derived exosomes on RAW264.7 cells. The experimental animals were male Sprague-Dawley (SD) rats aged 6–8 weeks, purchased from the Experimental Animal Center of Xinjiang Medical University. Each cage could house 4 rats. Housing conditions: the rats were kept in an environment with a temperature of 37 °C, a relative humidity of 50% ± 10%, and a 12-hour light/12-hour dark cycle. According to the above detection results, the exosome nucleic acid delivery system at a concentration of 10 μg/μL was used for intraperitoneal injection in SD rats to verify *in vivo* toxicity. Twenty-four hours later, rats in each group were anesthetized and euthanized via intravenous injection of pentobarbital injection at a dose of 100 mg/kg. Histological staining (HE) was performed to analyze pathological changes in major organs (heart, liver, spleen, lung, kidney) from different groups (Blank, Exo-miR155-ago, Exo-miR155-antago) for evaluating the *in vivo* toxicity of the exosome nucleic acid delivery system. Additionally, detections of liver and kidney functions as well as myocardial enzyme profiles were conducted.

### 2.5 Transcriptomic and proteomic analyses

#### 2.5.1 Sample collection and processing

Macrophage samples that had phagocytosed the exosome nucleic acid delivery system (10 μg/μL) were collected for transcriptomic and proteomic detections, respectively. For transcriptomic samples: cells were placed on ice, washed 3 times with pre-cooled PBS, lysed by adding TRIzol, then chloroform was added. After thorough oscillation and centrifugation, the aqueous phase was separated, and RNA was precipitated with pre-cooled isopropanol, followed by washing with absolute ethanol. Finally, total RNA was dissolved in enzyme-free DEPC water. For proteomic samples: the cells to be collected were placed on ice, washed 3 times with pre-cooled PBS, and pre-prepared cell lysis buffer was added for sufficient lysis to extract total proteins. After centrifugation at 4 °C for 5 min, the supernatant was collected as the total protein extract.

#### 2.5.2 Transcriptome sequencing and proteome quantitative analysis

After quality inspection of the RNA from each sample, transcriptome sequencing was performed. RNA libraries underwent sequencing at OE Biotech, Inc. located in Shanghai, China. For the mass spectrometry analysis of peptides, liquid chromatography–mass spectrometry was employed, with instruments from Thermo Scientific™ (Waltham, MA, USA), Orbitrap™ (Waltham, MA, USA), and Astral™ (Asheville, NC, USA). The relative quantification of proteins corresponding to the peptides was carried out by comparing the signal intensities of the relevant peptides across different samples. A quantitative analysis of the relative expression levels of proteins in various samples was accomplished through comparing information in the spectral library and integrating it into the corresponding proteins.

#### 2.5.3 Bioinformatics analysis

Gene Ontology (GO) enrichment analysis was conducted to explore the differences in differentially expressed genes mainly in the following three aspects: biological processes, cellular components, and molecular functions. Additionally, Kyoto Encyclopedia of Genes and Genomes (KEGG) enrichment analysis was performed to investigate their enrichment in different signaling pathways. These two enrichment analyses could further reveal the main biological functions and pathways involved in the changes in gene expression after macrophages were intervened by the exosome nucleic acid delivery system. For proteomic data, GO enrichment analysis and KEGG pathway analysis were also used to conduct comparative analysis of differentially expressed proteins. Specific parameter settings: the fold change was logarithmically transformed, i.e., FC, calculated by log_2_FC, where log_2_ (fold change) = mean value of the experimental group - mean value of the control group, and the p-value was obtained via t-test. Screening criteria for identification: p < 0.05 and FC ≥2.0.

### 2.6 Verification of macrophage autophagy and key signaling pathways

Macrophages were co-incubated with 10 μg/μL exosome nucleic acid delivery system and exosomes respectively for 12 h. Except for the negative control group, the culture medium of other experimental groups was replaced with EBSS medium for further incubation for 1 h to induce cell autophagy. Firstly, the number of autophagosomes and autolysosomes in cells of each group was observed by transmission electron microscopy (TEM) to evaluate the state of cell autophagy. Then, cells were stained using a cell autophagy staining detection kit, and the staining was observed under a confocal microscope followed by fluorescent quantitative analysis. Next, total proteins were extracted from cells of each group, and the expression levels of autophagy markers (LC3Ⅱ, p62, Beclin1) and autophagy-related pathway proteins (p-mTOR, p-AMPK, TLR4, p-p65, total mTOR, total AMPK, total p-p65) were detected by Western blotting, The method was the same as above. Details of the antibodies used are as follows: GAPDH (Sanying Biotechnology Co., Ltd., Wuhan, China; Cat. No.: #60004-1-Ig; Dilution ratio: 1:10000), LC3Ⅱ (Abclonal, Woburn, MA, USA; Cat. No.: #A19665; Dilution ratio: 1:1000), p62 (Abclonal, Woburn, MA, USA; Cat. No.: #A19700; Dilution ratio: 1:30000), p-mTOR (Sanying Biotechnology Co., Ltd., Wuhan, China; Cat. No.: #67778-1-IG; Dilution ratio: 1:5000), p-AMPK (Cell Signaling Technology, Danvers, MA, USA; Cat. No.: #2535S; Dilution ratio: 1:1000), p-p65 (Cell Signaling Technology, Danvers, MA, USA; Cat. No.: #3033; Dilution ratio: 1:1000), TLR4 (Sanying Biotechnology Co., Ltd., Wuhan, China; Cat. No.: #66350-1-Ig; Dilution ratio: 1:6000), Beclin1 (Sanying Biotechnology Co., Ltd., Wuhan, China; Cat. No.: #11306-1-AP; Dilution ratio: 1:8000). Finally, The method was the same as above, total RNA was extracted from cells of each group, and the mRNA expression levels of LC3B, p62, and Beclin1 were detected by RT-qPCR, It should be noted that a different reverse transcription kit, RevertAid First Strand cDNA Synthesis Kit (Thermo Fisher Scientific, Waltham, MA, USA, Cat. No.: K1622), was used for reverse transcription at this step. (Primer sequences are shown in Table 1).

### 2.7 Statistical analysis

All experiments were independently repeated three times. Data were statistically analyzed using GraphPad Prism 9.0 software, and image composition was performed using Adobe Illustrator 2020 and Adobe Photoshop 2023 software. Quantitative data are presented as “mean ± SD”, and comparisons between groups were conducted using the t-test or one-way analysis of variance (one-way ANOVA). A P-value <0.05 was considered to indicate a statistically significant difference.

## 3 Results

### 3.1 Extraction of exosomes, construction and characterization of exosome nucleic acid delivery system

**FIGURE 2:**
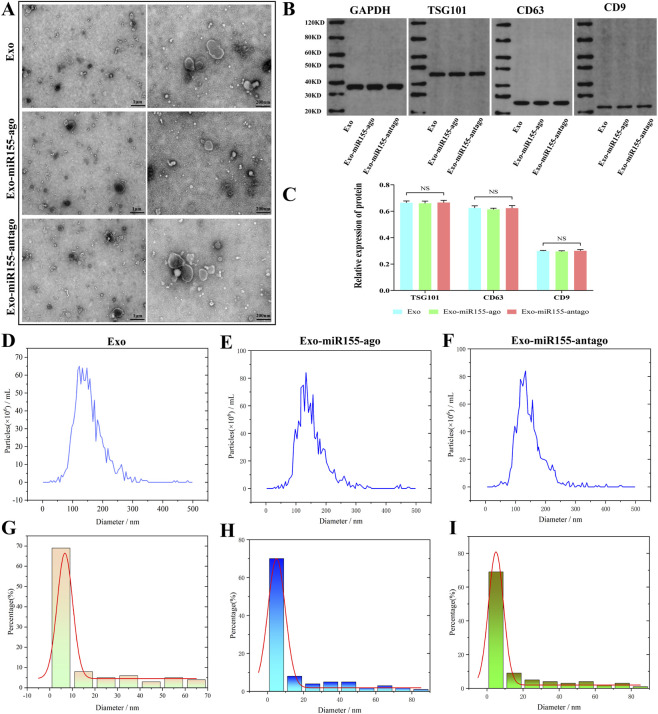
Construction and characterization of exosome nucleic acid delivery system (Exo-miR155-ago/Exo-miR155-antago). **(A)** Transmission electron microscopy (TEM) showing bone marrow mesenchymal stem cell exosomes (Exo) and exosome nucleic acid delivery systems; scale bars, 1μm, 200 nm. **(B,C)** Western blot results before and after exosome transfection (NS, Not Significant). **(D–I)** Nanoparticle tracking analysis (NTA), particle size frequency distribution and Gaussian distribution of bone marrow mesenchymal stem cell exosomes and exosome nucleic acid delivery systems.

Transmission electron microscopy (TEM) observation revealed that exosomes before and after transfection exhibited a typical double-membrane structure, with a cup-shaped or circular morphology. Exosome particles in each sample group varied in size, and the morphology of exosome membranes remained unchanged, indicating that the construction of the exosome nucleic acid delivery system had no impact on the morphological structure of exosomes (Figure 2A). Western blotting (WB) results showed no significant differences in the relative expression levels of exosome-specific proteins among the sample groups (Figures 2B,C), suggesting that the composition of exosomes was not significantly affected. Nanoparticle tracking analysis (NTA) demonstrated that the particle size distribution of exosomes presented a unimodal normal distribution pattern. The particle size distribution of each sample group was approximately 30–200 nm, with the highest frequency around 130 nm (Figures 2D–F). Additionally, the particle size frequency distribution and Gaussian distribution plots confirmed that the extracted particles conformed to the size characteristics of exosomes, indicating that the construction of the exosome nucleic acid delivery system did not alter the structure of exosomes (Figures 2G–I).

### 3.2 Verification of construction and phagocytosis efficiency of exosome nucleic acid delivery system

**FIGURE 3:**
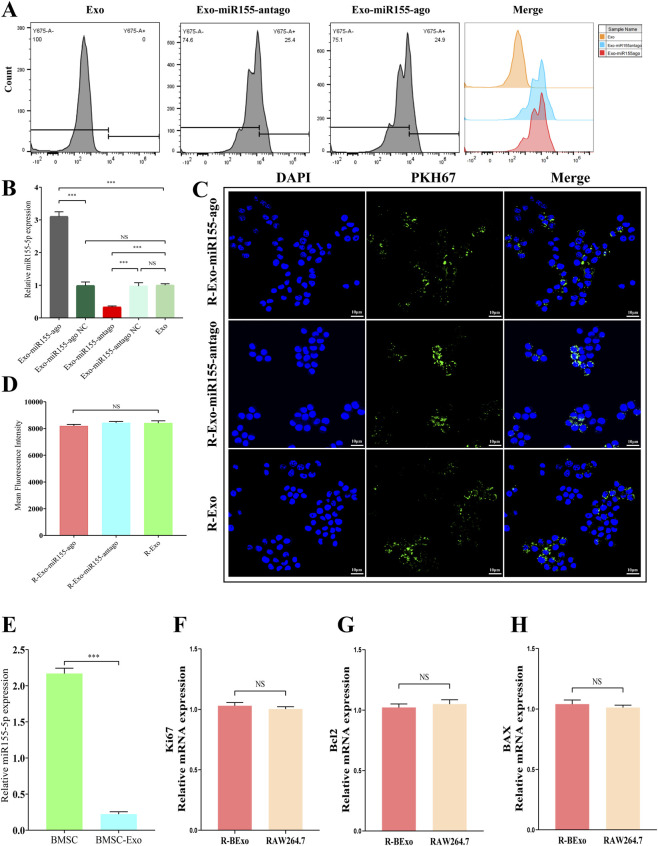
Detection of loading efficiency of exosome nucleic acid delivery system (Exo-miR155-ago/Exo-miR155-antago). **(A)** Flow cytometry analysis of transfection efficiency of exosomes loaded with Cy5 fluorescently labeled miR155-5P agonists and inhibitors. **(B)** RT-qPCR analysis of miR155-5P expression in exosome nucleic acid delivery systems, Among them, Exo-miR155-ago NC represents the negative control group of miR-155-agomir, and Exo-miR155-antago NC represents the negative control group of miR-155-antagomir (NS, Not Significant; ***, p < 0.01). **(C)** Confocal microscopy images showing fluorescence expression in macrophages after co-incubation of exosome nucleic acid delivery systems with macrophages; cell nuclei are labeled with DAPI (blue), and exosomes are labeled with PKH 67 (green); scale bar, 10 μm. **(D)** Quantitative analysis of PKH 67 mean fluorescence intensity. (NS, Not Significant). **(E)** Quantitative real-time polymerase chain reaction (RT-qPCR) was performed to detect the content of endogenous miR155-5p in bone marrow mesenchymal stem cells (BMSCs) and their exosomes (***, p < 0.01, indicating a significant difference); **(F–H)** Quantitative real-time polymerase chain reaction (RT-qPCR) was employed to determine the proliferation (relative mRNA expression level of Ki67) and apoptosis (relative mRNA expression level of BCL2/BAX) of RAW264.7 cell line in both the co-culture system of exosomes alone with RAW264.7 cell line and the RAW264.7 cell line itself. This experiment aimed to clarify the effect of endogenous miR155-5p on cell functions (NS, no significant difference).

Flow cytometry results showed that the transfection efficiency of the Exo-miR155-ago group was 24.9%, and that of the Exo-miR155-antago group was 25.4%. Compared with previous methods such as electroporation or sonication, transfection using a dedicated kit significantly improved the transfection efficiency (Figure 3A). RT-qPCR results showed that the content of miR155-5p in the experimental group was significantly higher than that in the control group, indicating the successful construction of the exosome nucleic acid delivery system (Figure 3B). In addition, to evaluate the potential impact of endogenous miR155 - 5p on functional experiments, we detected the level of miR155 - 5p in BMSCs and their secreted exosomes via RT - qPCR, which revealed that the content of miR155 - 5p in exosomes was far lower than that in BMSCs. This indicated that the level of endogenous miR155 - 5p in exosomes was extremely low compared with that in the parent BMSCs. Thus, the exosome nucleic acid delivery system constructed by us served as the primary source of cellular functional effects, effectively eliminating the interference from endogenous background (Figure 3E). Confocal microscopy observation of macrophage phagocytosis of exosomes showed that the exosome nucleic acid delivery system had no significant inhibitory effect on macrophage uptake of exosomes, which was consistent with the results of fluorescent quantitative analysis (Figures 3C,D). Finally, to functionally verify the potential impact of endogenous miR155-5p at the cellular level, we set up the experimental group (R-BExo) by co-culturing untransfected naive exosomes with macrophages, and used macrophages alone as the control group (RAW264.7). We detected macrophage proliferation (Ki67) and apoptosis (BCL2/BAX) via RT-qPCR. Compared with the control group, the untransfected naive exosome group exhibited no statistically significant differences in the mRNA expression levels of the proliferation-related gene Ki67 and the apoptosis-related genes BCL2/BAX (Figures 3F–H).

### 3.3 Verification of in vitro/in vivo toxicity of exosome nucleic acid delivery system

**FIGURE 4:**
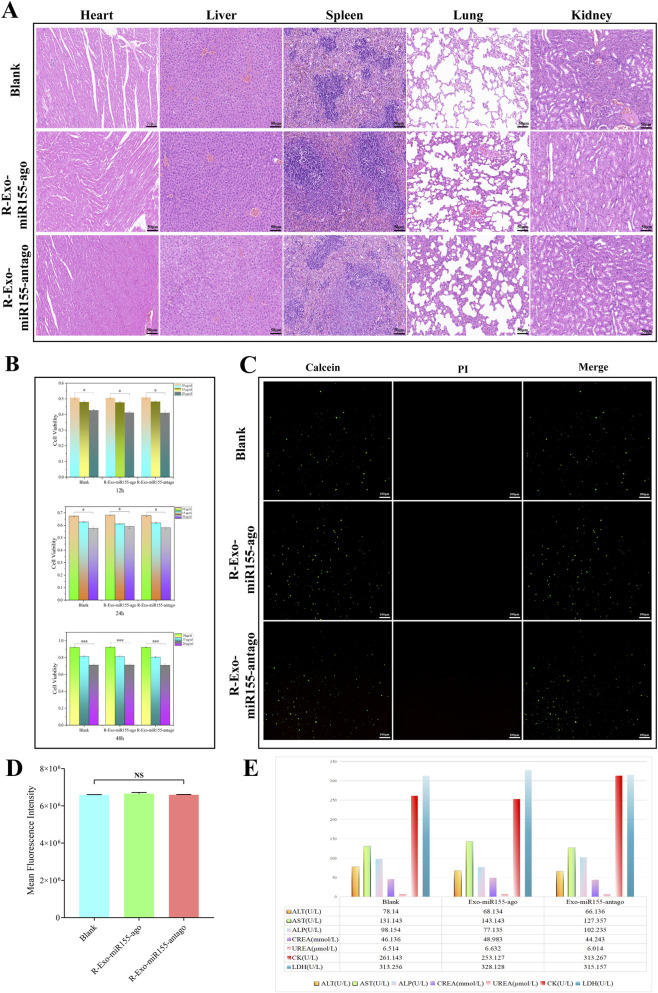
*In vivo*/*in vitro* toxicity verification of exosome nucleic acid delivery system (Exo-miR155-ago/Exo-miR155-antago). **(A)** Hematoxylin and eosin (HE) staining of major organs; scale bar, 50 μm. **(B)** Effect of different concentrations on RAW264.7 cell viability, determined by CCK-8 assay. **(C)** Live/dead staining of RAW264.7 cell line with 10 μg/μL exosome nucleic acid delivery system; scale bar, 100 μm. **(D,E)** Detection of kidney/liver function and myocardial enzyme levels. Among them, the prefix “R” in Panels **(A–D)** stands for the RAW264.7 cell line, which is used to indicate the state of the exosome nucleic acid delivery system after co-cultivation with macrophages (*, p ≤ 0.05; ***, p ≤ 0.01; NS, Not Significant).

CCK-8 assay results showed that the exosome nucleic acid delivery system at a concentration of 10 μg/μL had the least impact on macrophage viability (Figure 4B). Subsequent cell live/dead staining and fluorescent quantitative analysis at this concentration showed small differences among the experimental groups (Figures 4C,D). In HE staining of SD rat tissues, no pathological changes indicating organ damage were detected (Figure 4A). Moreover, laboratory tests showed that liver function, kidney function, and myocardial enzyme levels were all within the normal range (Figure 4E).

### 3.4 Transcriptomic results

#### 3.4.1 Differential gene expression analysis

**FIGURE 5:**
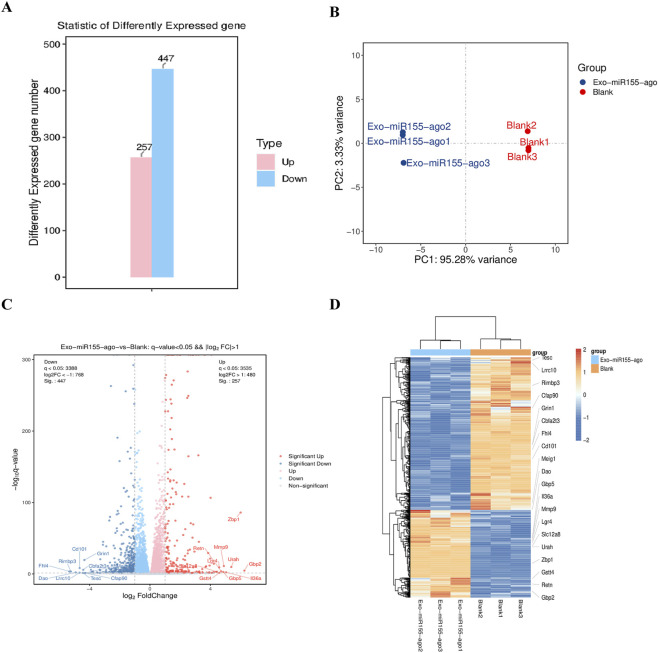
Transcriptomic analysis of macrophages after intervention with exosome nucleic acid delivery system (Exo-miR155-ago). **(A)** Comparison of expression levels of upregulated and downregulated differential genes. **(B)** Principal component analysis (PCA). **(C)** Volcano plot of differential gene expression, where upregulated genes are distributed in the right area of the plot and downregulated genes are distributed in the left area; each dot represents a gene, and the size of each dot represents the strength of the differential gene. **(D)** Clustered heatmap of differential gene groups. Orange-yellow indicates relatively highly expressed coding genes, and blue indicates relatively lowly expressed coding genes.

Transcriptomic analysis revealed that the expression of 704 genes changed significantly, including 257 upregulated genes and 447 downregulated genes (Figure 5A). Principal component analysis (PCA) of each sample showed significant differences between the two groups, with PC 1 explaining 95.28% of the variance and PC 2 explaining 3.33% (Figure 5B). A volcano plot was generated for differentially expressed genes to visually summarize the overall changes in gene expression (Figure 5C). Finally, a heatmap was plotted for the experimental and control groups to facilitate a more intuitive analysis of differentially expressed genes, where red indicates relatively highly expressed protein-coding genes and blue indicates relatively lowly expressed ones (Figure 5D). Among them, inflammation-related genes and immune-related genes showed high significance in expression differences, indicating that treatment with the exosome nucleic acid delivery system had a significant impact on macrophage immune regulation and inflammation.

#### 3.4.2 Gene enrichment analysis

**FIGURE 6:**
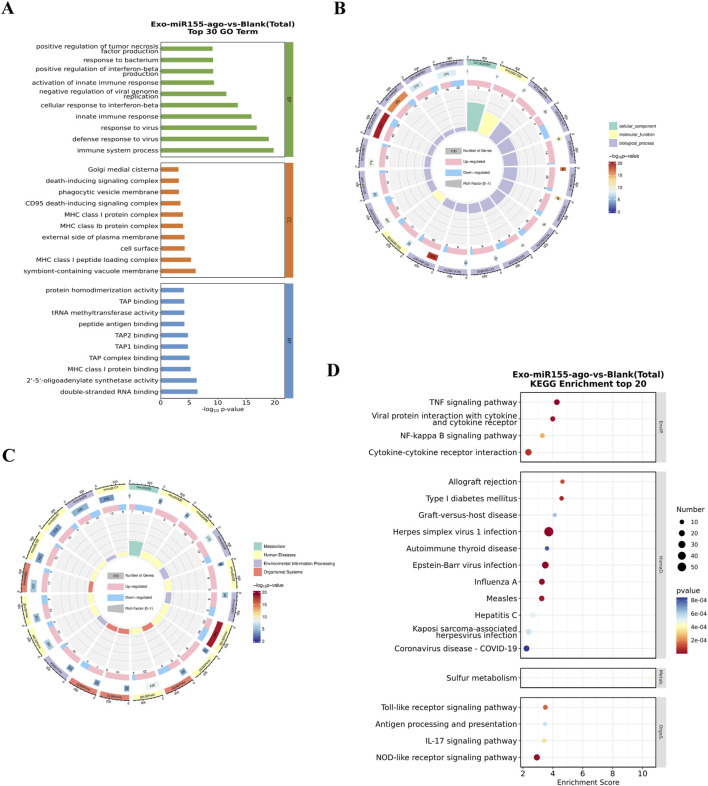
**(A)** Top 30 bar charts of GO enrichment analysis in the transcriptomics of macrophages after intervention with the exosome nucleic acid delivery system (Exo-miR155-ago) (screening GO terms with PopHits ≥5 in the three categories, with 10 terms each sorted by -log10p-value of each term in descending order) and **(B)** circular plots. **(C)** Circular plots of KEGG enrichment analysis and **(D)** bubble plots of the top 20 Kyoto Encyclopedia of Genes and Genomes (KEGG) pathway enrichments.

Gene Ontology (GO) enrichment analysis showed that these differentially expressed genes were significantly enriched in three aspects: biological processes, cellular components, and molecular functions. Biological processes were mainly concentrated in “immune system process,” “defense response to virus,” “response to virus,” “innate immune response,” and “cellular response to interferon-beta.” Cellular components were mainly enriched in “symbiont-containing vacuole membrane,” “MHC class I peptide loading complex,” “cell surface,” “external side of plasma membrane,” and “MHC class Ib protein complex”, among which GO:0016020 (membrane, PopHits = 7693) was the most significant, suggesting that the exosome nucleic acid delivery system may have altered the structure or composition of cell membranes. Molecular functions were mainly focused on “double-stranded RNA binding”, “2-5-oligoadenylate synthetase activity,” “MHC class I protein binding,” and “TAP complex binding,” with GO:0005515 (protein binding, PopHits = 5380) being the most significant, indicating that the system may have changed intracellular protein-binding capacity. These functions are closely related to macrophage recognition and uptake of exosomes, as well as subsequent signal transduction and functional regulation (Figures 6A,B).

KEGG enrichment analysis showed significant changes in pathways such as “TNF signaling pathway,” “Viral protein interaction with cytokine and cytokine receptor,” “NF-κB signaling pathway,” and “Cytokine-cytokine receptor interaction.” Among them, the TNF signaling pathway had the most significant differentially expressed genes, indicating that a large number of differentially expressed genes were involved in this process after macrophages phagocytosed the exosome nucleic acid delivery system. These most significantly enriched pathways are closely related to cell autophagy (Zhou et al., 2018; Yang et al., 2022), further suggesting that the exosome nucleic acid delivery system directly or indirectly affects signaling pathways such as TNF/NF-κB, thereby influencing cell fate in biological processes such as immune regulation, inflammatory response, and cell autophagy, ultimately achieving effective activation of macrophage immune-inflammatory pathways by the exosome nucleic acid delivery system (Figures 6C,D).

### 3.5 Proteomic sequencing results

#### 3.5.1 Differential protein expression analysis

**FIGURE 7:**
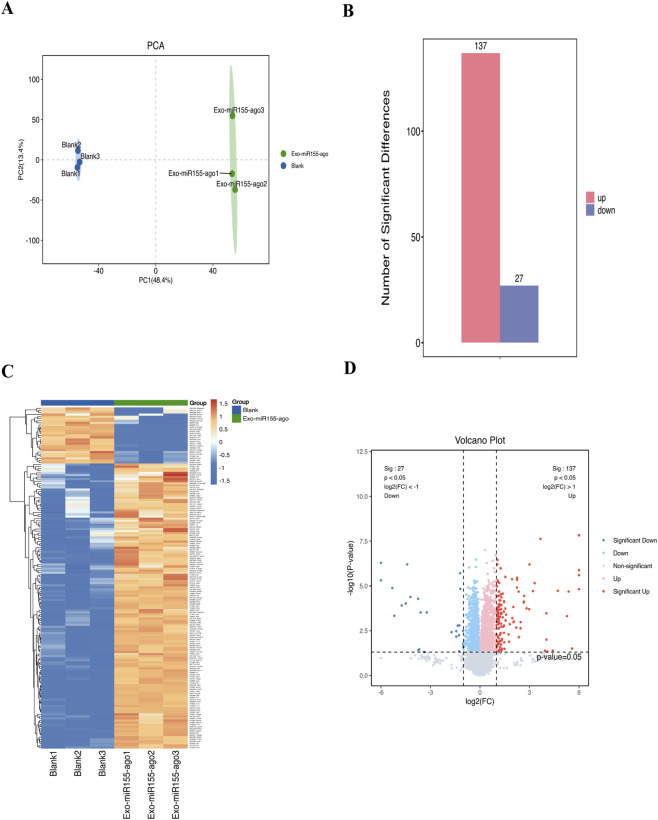
Proteomic analysis of macrophages after intervention with the exosome nucleic acid delivery system (Exo-miR155-ago). **(A)** Principal component analysis (PCA):The results showed that the two groups of samples exhibited a clear tendency of separation in the dimensions where the contribution rate of PC1 was 48.4% and that of PC2 was 13.4%. Moreover, the 3 biological replicate samples within the same group were closely clustered, indicating significant overall differences in the protein expression profiles of macrophages between the two groups and verifying the reliability of experimental replicates. **(B)** Comparison of expression levels of upregulated and downregulated differential proteins: A total of 164 proteins changed, including 137 upregulated proteins and 27 downregulated proteins. **(C)** Clustered heatmap of differentially expressed proteins, Hierarchical clustering analysis was performed based on the expression levels of 164 significantly differentially expressed proteins in the two groups of samples, so as to intuitively display the inter-group differences in protein expression patterns. In the heatmap, rows represent individual differentially expressed proteins, and columns represent different samples (intervention group/control group, with 3 replicates in each group); the color gradient is set such that orange-yellow indicates high protein expression and blue indicates low protein expression. **(D)** Volcano plot of differentially expressed proteins; With log_2_FC as the abscissa (reflecting the fold change in protein expression) and -log_10_(P-value) as the ordinate (reflecting the significance of differences), it displays the distribution characteristics of 164 significantly differentially expressed proteins. In the plot, red dots represent significantly upregulated differentially expressed proteins (total of 137), blue dots represent significantly downregulated differentially expressed proteins (total of 27), and the darker the color, the more significant the difference (i.e., larger |log_2_FC| values and smaller P-values); gray dots represent non-significantly differentially expressed proteins with a P-value ≥0.05.

Data-independent acquisition (DIA) showed that PCA revealed significant differences between the two groups, with PC 1 explaining 48.4% of the variance and PC 2 explaining 13.4% (Figure 7A). A total of 164 proteins changed, including 137 upregulated proteins and 27 downregulated proteins (Figure 7B). Heatmaps and volcano plots were generated for these differentially expressed proteins to more intuitively show the overall changes in protein expression (Figures 7C,D).

#### 3.5.2 Protein enrichment analysis

**FIGURE 8:**
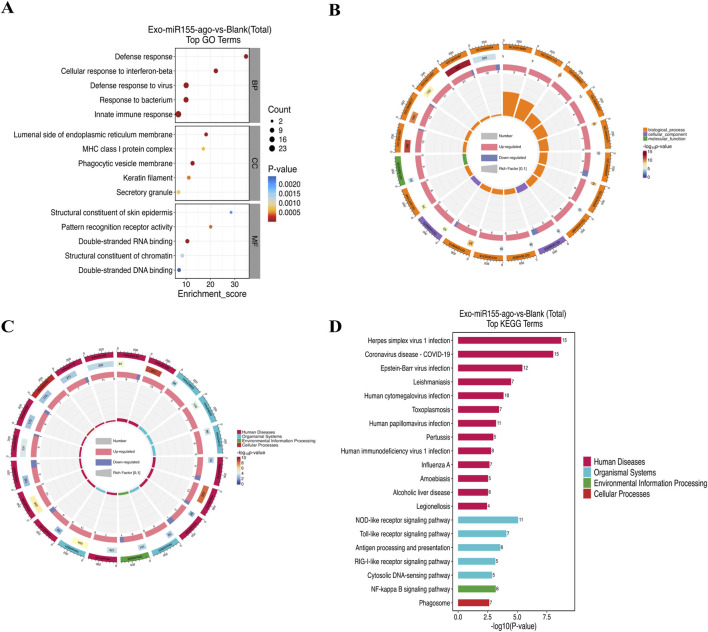
**(A)** Bubble plot and **(B)** circular plot of GO enrichment in the proteomic analysis of macrophages after intervention with the exosome nucleic acid delivery system (Exo-miR155-ago). **(C)** Circular plot of KEGG enrichment analysis and **(D)** top 20 bar chart, where the horizontal axis represents the number of foreground proteins in each term, and the vertical axis represents the term/pathway name.

GO enrichment analysis revealed significant changes in biological processes, cellular components, and molecular functions. In terms of biological processes, “defense response,” “cellular response to interferon-beta,” “defense response to virus,” “response to bacterium”, and “innate immune response” were significantly altered. For cellular components, “lumenal side of endoplasmic reticulum membrane,” “MHC class I protein complex,” “phagocytic vesicle membrane,” “keratin filament,” and “secretory granule” showed significant changes. In molecular functions, “structural constituent of skin epidermis,” “pattern recognition receptor activity,” “double-stranded RNA binding,” “structural constituent of chromatin,” and “double-stranded DNA binding” were significantly changed. These results are consistent with the transcriptomic findings, further indicating that the exosome nucleic acid delivery system can enhance the immune defense function of macrophages, thereby promoting processes such as cell autophagy and ultimately playing a role in resisting pathogen infections such as Mtb (Figures 8A,B).

KEGG enrichment analysis in proteomics showed a significant change in the “NF-κB signaling pathway,” which was consistent with the transcriptomic results, re-emphasizing the importance of the NF-κB signaling pathway in this study. In the “Cellular Processes” category, pathways related to “Phagosome” changed significantly. Phagosomes are structures formed by macrophages after phagocytosing pathogens, and their maturation and fusion with lysosomes are key steps in pathogen clearance (Uribe-Querol and Rosales, 2020; Liu et al., 2017). The exosome nucleic acid delivery system may affect the formation, maturation, and fusion of phagosomes with lysosomes by regulating the expression of phagosome-related proteins, thereby influencing macrophage autophagy. NOD-like receptors and Toll-like receptors are important members of the pattern recognition receptor family, which can recognize pathogen-associated molecular patterns (PAMPs) and activate downstream immune signaling pathways, including the NF-κB signaling pathway, thereby regulating immune responses and autophagy (Tang et al., 2012; Zhu et al., 2019). These signaling pathways are interconnected, forming a complex regulatory network. The exosome nucleic acid delivery system may synergistically enhance macrophage autophagy by regulating these signaling pathways, improving the body’s immune defense against Mtb (Figures 8C,D).

### 3.6 Integrated analysis of transcriptomics and proteomics

**FIGURE 9:**
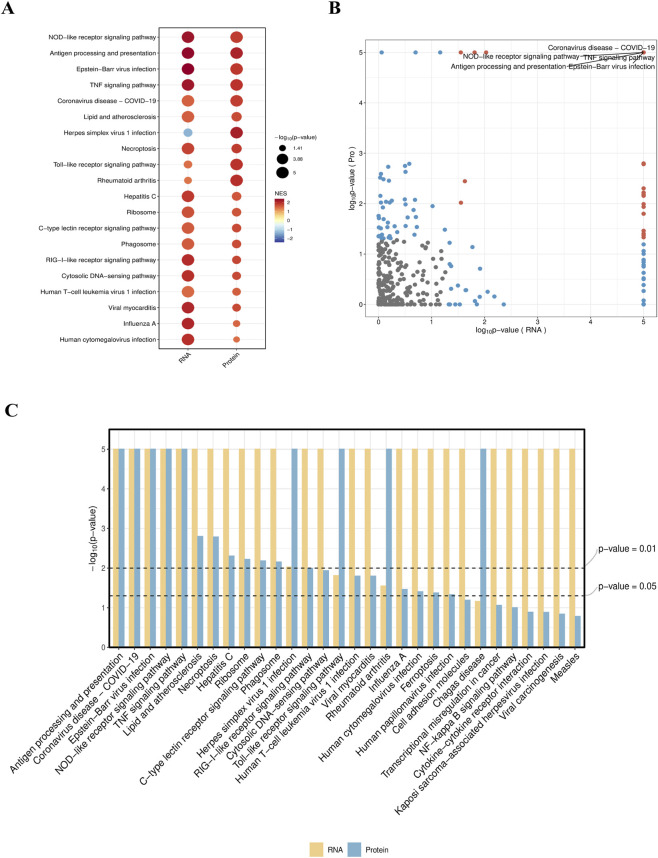
Integrated analysis of transcriptomics and proteomics of macrophages after intervention with the exosome nucleic acid delivery system (Exo-miR155-ago). **(A)** Bubble plot: Displays the functionally co-enriched pathways between the two omics datasets. The x-axis represents the pathway enrichment multiple, the y-axis represents pathway names, and the size of bubbles indicates the number of differential molecule pairs in the pathway. The darker the color (from red to blue), the smaller the enrichment P-value (and the higher the significance). The results show that the bubbles for autophagy and inflammation-related signaling pathways are the largest and darkest (P < 0.01), which are the core regulatory pathways, **(B)** Volcano plot: Simultaneously presents the distribution of differential RNAs and differential proteins. Red represents co-upregulated molecules, blue represents co-downregulated molecules, and gray represents molecules with no significant differences. The results show that the NOD-like receptor signaling pathway, TNF signaling pathway, and antigen processing and presentation pathway showed the most significant changes, and the two omics datasets exhibit high synergy, **(C)** Bar chart: t compares the relative expression levels of key molecules in core pathways (including autophagy, inflammatory pathways, and NOD-like receptor signaling pathway) between RNAs (light yellow bars) and proteins (light blue bars). Error bars represent SD, with baseline markers for P = 0.05 and P = 0.01 respectively. The expression trends of transcriptomics and proteomics are consistent.

KEGG integrated enrichment analysis was performed on key pathways regulated by differentially expressed genes and proteins, and bubble plots, volcano plots, and bar graphs were generated. Among them, the NOD-like receptor signaling pathway, TNF signaling pathway, and antigen processing and presentation pathway showed the most significant changes. The NOD-like receptor pathway, as a core pathway for intracellular pathogen recognition, can initiate inflammasome assembly and cytokine release when activated. The significant changes in the TNF signaling pathway strengthen the inflammatory cascade and immune cell recruitment. Abnormalities in the antigen processing and presentation pathway directly affect the efficiency of MHC molecule-mediated antigen presentation. Together, they suggest that the exosome nucleic acid delivery system may participate in the regulatory network of enhanced macrophage autophagy by strengthening pathogen recognition, inflammatory regulation, and adaptive immune activation (Figures 9A–C).

### 3.7 Verification of macrophage autophagy and related pathways

To validate the macrophage autophagy regulatory mechanism revealed by previous transcriptomics and proteomics studies, multi-dimensional experiments were subsequently conducted to detect autophagy-related indicators and the expression of key signaling molecules.

**FIGURE 10:**
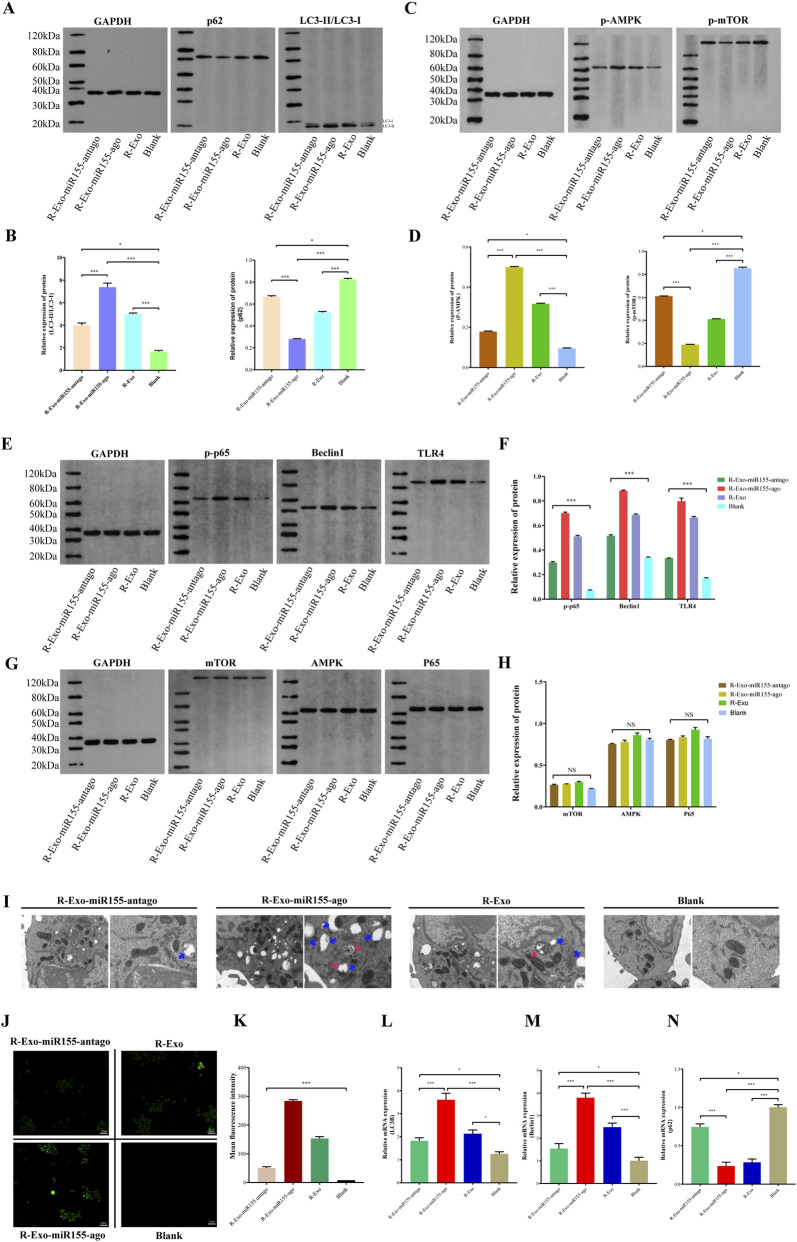
Verification of autophagy levels and related signaling pathways in macrophages after intervention with the exosome nucleic acid delivery system (Exo-miR155-ago/Exo-miR155-antago). **(A,B)** Western blot (WB) analysis of LC3 and p62 protein expression and their relative expression levels. **(C,D)** Western blot (WB) analysis of phosphorylated protein expression in the mTOR/AMPK signaling pathway and their relative expression levels. **(E,F)** Western blot (WB) analysis of p-p65, TLR4, and Beclin1 protein expression and their relative expression levels. **(G,H)** Western blot (WB) analysis was performed to detect the expression and relative expression levels of total proteins of mTOR, AMPK, and P65; **(I)** Transmission electron microscopy (TEM) was used to observe the cellular autophagy morphology of each group, with scale bars of 1 μm and 500 nm (red arrows indicate autophagosomes, and blue arrows indicate autolysosomes); **(J,K)** Monodansylcadaverine (MDC) staining was adopted to detect cellular autophagy levels, followed by quantitative analysis of fluorescence intensity, with a scale bar of 25 μm; **(L–N)** Quantitative real-time polymerase chain reaction (RT-qPCR) was conducted to determine the mRNA expression levels of autophagy-related genes LC3B/p62/Beclin1. The prefix “R” in Panel 10 stands for the RAW264.7 cell line, which is used to indicate the state of the exosome nucleic acid delivery system after co-cultivation with macrophages (*, p ≤ 0.05; ***, P ≤ 0.01).

At the level of autophagy phenotype verification, transmission electron microscopy observations showed that the number of autophagosomes and autolysosomes in macrophages of the R-Exo-miR155-ago group was significantly higher than that in the control group. This morphological change was directly consistent with the results of phagosome pathway activation and changes in the expression of autophagy-executing proteins in the previous proteomics, confirming the enhancement of autophagy activity at the subcellular structure level (Figure 10I). Meanwhile, MDC staining combined with fluorescence quantification revealed that the cells in the R-Exo-miR155-ago group showed the most obvious staining, intuitively reflecting the increased enrichment of autophagic vacuoles. This further verified the conclusion from transcriptomic analysis that autophagy-related biological processes were significantly enriched, providing evidence for autophagy activation from the perspective of functional phenotype (Figures 10J,K).

At the level of verifying the expression of autophagy-related molecules, RT-qPCR results showed that the mRNA expressions of autophagy-promoting genes LC3B and Beclin1 were upregulated, while the mRNA expression of the autophagy substrate gene p62 was downregulated (Figures 10L–N). This gene expression trend was completely consistent with the results of Western blot (WB) detection: WB showed high expressions of LC3 protein (especially LC3Ⅱ) and Beclin1 protein, and low expression of p62 protein in the R-Exo-miR155-ago group (Figures 10A,B). More importantly, this consistency in expression from the transcriptional to the translational level not only echoes the differential expression patterns of the above genes in transcriptomics but also corroborates the results of changes in the expression of autophagy initiation-related proteins in proteomics, clarifying the expression rules of autophagy regulation at the molecular level.

At the level of verifying key autophagy signaling pathways, further WB detection found that in the R-Exo-miR155-ago group, the expression of p-mTOR, a core molecule of the energy-sensing pathway, was decreased, while the expression of p-AMPK was increased. This change conforms to the classical mechanism by which the AMPK/mTOR pathway regulates autophagy and forms a logical closed loop with the results of transcriptomics showing that TNF signaling pathway activation induces metabolic stress (Figures 10C,D). In addition, WB detection results and quantitative analysis indicated that the expressions of pathogen recognition receptor TLR4, downstream NF-κB pathway key molecule p-p65, and autophagy-related protein Beclin1 were sequentially increased in the R-Exo-miR155-ago group (Figures 10E,F). This trend of signal activation was highly consistent with the results of transcriptomics and proteomics showing significant activation of the NOD-like receptor signaling pathway and NF-κB pathway. The above pathway changes together construct a complete molecular evidence chain of pathogen recognition → signal transduction → autophagy initiation, ultimately clarifying that the exosomal nucleic acid delivery system can enhance macrophage autophagy activity by synergistically activating the two core pathways of TLR4/NF-κB and AMPK/mTOR. To rule out the influence of changes in the total expression levels of AMPK/mTOR/P65 proteins on their phosphorylation levels among different groups, we further detected the total protein levels of AMPK/mTOR/P65 in each experimental group. As shown in the figure, the total protein expression levels of AMPK/mTOR/P65 remained stable in all experimental groups, with no significant differences observed among the groups. This result indicates that the previously observed changes in the phosphorylation levels of AMPK/mTOR/P65 (p-AMPK/p-mTOR/p-P65) are dominated by alterations in their phosphorylation status rather than fluctuations in their total protein expression levels (Figures 10G,H).

## 4 Discussion

The core pathological contradiction of tuberculosis (TB) lies in the fact that *Mycobacterium tuberculosis* (Mtb) can achieve intracellular immune escape by inhibiting macrophage autophagy. Specifically, Mtb interferes with the fusion of autophagosomes and lysosomes, blocks the autophagic degradation process, and ultimately colonizes and proliferates within macrophages, leading to persistent infection (Deretic et al., 2015). As a key defense mechanism for the host to clear intracellular Mtb, the regulation of autophagic activity directly determines the anti-TB capacity of macrophages (Yu et al., 2024). MicroRNA-155 (miR-155) is precisely the core molecule in this regulatory process. Existing studies have confirmed that miR-155 can regulate macrophage polarization, inflammatory responses, and autophagic activity by targeting relevant signaling pathways (Ghafouri-Fard et al., 2021). Moreover, its expression level changes significantly during Mtb infection, suggesting that it may serve as a key target for remodeling the anti-TB function of macrophages (Yu et al., 2022). Based on this, targeted regulation of miR-155 expression in macrophages to activate autophagy has become an important direction for overcoming Mtb immune escape and developing novel anti-TB strategies, which also provides a core logical basis for the subsequent experimental design of this study.

This study first constructed a novel RNA delivery system and performed a series of characterizations. Subsequently, from three aspects—autophagy phenotype observation, molecular expression detection, and signaling pathway verification—transcriptomics and proteomics were used to systematically validate changes in the expression of autophagy-related indicators and key signaling molecules. Observations of subcellular structures via transmission electron microscopy (TEM) showed that the number of autophagosomes and autolysosomes in macrophages of the R-Exo-miR155-ago group was significantly higher than that in the control group. This change in morphological characteristics is not an isolated phenomenon but directly consistent with the results of proteomic analysis, which revealed significant activation of the phagosome pathway and upregulated expression of autophagy-executing proteins. The activation of the phagosome pathway in proteomics suggests enhanced phagosome formation and maturation, while changes in the expression of autophagy-executing proteins provide a molecular basis for the fusion of autophagosomes with lysosomes (Piscitelli et al., 2024). The increased number of autophagosomes observed by TEM is an intuitive manifestation of these molecular changes at the subcellular structural level, further confirming the enhancement of autophagy activity from a morphological perspective.

Meanwhile, MDC staining experiments showed that cells in the experimental group exhibited the most obvious autophagy-specific staining, reflecting a significant increase in the enrichment of intracellular autophagic vacuoles. This result also echoes the conclusion from transcriptomic analysis that autophagy-related biological processes are significantly enriched: transcriptomics reveals the activation trend of autophagy-related biological processes through differential gene expression, while MDC staining directly demonstrates the actual state of autophagic vacuole accumulation through functional detection. Together, these two approaches—from the prediction of molecular processes to the verification of functional phenotypes—support the core view that the exosomal nucleic acid delivery system can activate macrophage autophagy.

RT-qPCR results showed that the mRNA expressions of LC3B and Beclin1 were significantly upregulated, while the mRNA expression of p62 was significantly downregulated. More importantly, this gene expression trend was confirmed by Western blot (WB) experiments: WB detection showed high expressions of LC3 and Beclin1 proteins in the experimental group, while the expression of p62 protein, an autophagic degradation substrate, was low. This consistency in expression from the transcriptional to the translational level not only highly corresponds to the differential expression patterns of LC3B, Beclin1, and p62 genes in transcriptomic analysis but also further confirms that the regulation of these genes at the transcriptional level is not accidental. Such cross-omics and cross-level consistency of results greatly enhances the reliability of the view that the exosomal nucleic acid delivery system enhances autophagy by regulating the expression of key molecules.

Finally, this study verified the activation of key pathways at the protein level. WB experiments further revealed the activation patterns of two core pathways, both of which form a logical closed loop with previous omics results. On the one hand, the expression of core molecules in the energy-sensing pathway in the experimental group was characterized by decreased p-mTOR and increased p-AMPK. This change fully conforms to the classical mechanism by which the AMPK/mTOR pathway regulates autophagy: AMPK activation can inhibit mTOR activity through phosphorylation, thereby relieving the negative regulation of autophagy by mTOR. More critically, this pathway change is logically related to the results of previous transcriptomics showing that TNF signaling pathway activation induces cellular metabolic stress: the inflammatory response triggered by TNF signaling pathway activation increases cellular energy consumption, which in turn triggers AMPK-mediated energy-sensing regulation, and ultimately promotes autophagy initiation by inhibiting mTOR, forming a complete logical chain of inflammatory stress → metabolic regulation → autophagy activation. (p- denotes phosphorylation, a key post-translational modification regulating protein activity)

**FIGURE 11:**
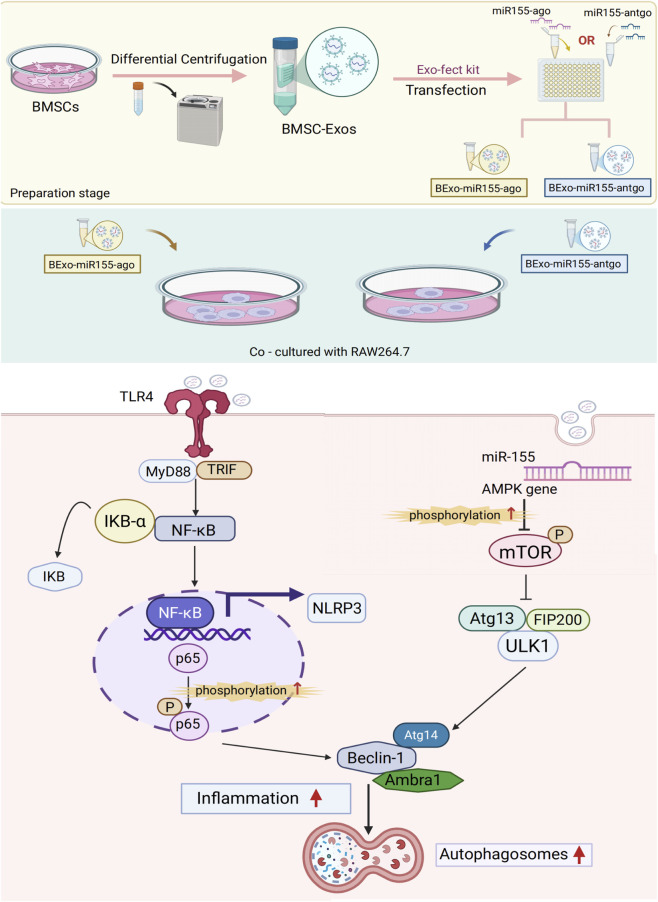
Schematic diagram of exosomal nucleic acid delivery system bidirectionally driving macrophage autophagic activity by synergistically activating TLR4/NF-κb signaling pathway and AMPK/mTOR Pathway (by biorender).

On the other hand, the expressions of the pathogen recognition receptor TLR4 and the key downstream NF-κB pathway molecule p-p65 in the experimental group showed a sequential increase. This signal activation is highly consistent with the results of transcriptomics and proteomics showing significant activation of the NOD-like receptor signaling pathway and NF-κB pathway. As a key receptor for recognizing pathogen-associated molecular patterns (PAMPs) of *Mycobacterium tuberculosis* (Mtb), the upregulated expression of TLR4 indicates enhanced pathogen recognition ability of macrophages (Mortaz et al., 2017; Sepehri et al., 2019). The activation of downstream p-p65 directly confirms the functional activation of the NF-κB pathway, which forms a complete with the differential expression of NF-κB target genes (such as Beclin1) in transcriptomics and changes in the expression of NF-κB pathway-related proteins in proteomics, clarifying the molecular pathway of pathogen recognition → TLR4 activation → NF-κB signal transduction → transcription of autophagy-related genes (Figure 11).

## 5 Conclusion

The core pathological contradiction of tuberculosis (TB) lies in the fact that *Mycobacterium tuberculosis* (Mtb) achieves immune escape by inhibiting macrophage autophagy and causes persistent infection. Autophagy is the host’s key mechanism for clearing intracellular Mtb, while miR-155, as a core molecule regulating autophagy, can serve as an anti-TB target and provides the core logical basis for this study.

In this study, a novel exosomal nucleic acid delivery system was constructed. By integrating transcriptomics, proteomics, and cellular validation experiments, we systematically investigated the immune remodeling mechanism of Exo-miR155-ago in macrophage autophagy from three dimensions: autophagic phenotype, molecular expression, and signaling pathways. The results confirmed that this system can dually drive the enhancement of macrophage autophagy activity by synergistically activating the TLR4/NF-κB signaling pathway (strengthening pathogen recognition and the transcription of autophagy-related genes) and the AMPK/mTOR pathway (regulating energy metabolism to relieve autophagy inhibition). Although this study did not employ Mtb-infected macrophages, the exosomal nucleic acid delivery system may provide potential possibilities for the development of novel therapeutic agents against *Mycobacterium tuberculosis* infection.

## Data Availability

Transcriptomic raw data: Genome Sequence Archive (GSA), China National Center for Bioinformation/Beijing Institute of Genomics, Chinese Academy of Sciences, accession number CRA029638, publicly accessible at https://ngdc.cncb.ac.cn/gsa. Proteomic raw data: OMIX, China National Center for Bioinformation/Beijing Institute of Genomics, Chinese Academy of Sciences, accession number OMIX011766, publicly accessible at https://ngdc.cncb.ac.cn/omix.
